# The Immediate and Late Effects of Thyroid Hormone (Triiodothyronine) on Murine Coagulation Gene Transcription

**DOI:** 10.1371/journal.pone.0127469

**Published:** 2015-05-26

**Authors:** Salam Salloum-Asfar, Anita Boelen, Pieter H. Reitsma, Bart J. M. van Vlijmen

**Affiliations:** 1 Einthoven Laboratory for Experimental Vascular Medicine, Department of Thrombosis and Hemostasis, Leiden University Medical Center, Leiden, the Netherlands; 2 Department of Hematology and Medical Oncology, Centro Regional de Hemodonación, IMIB-Arrixaca, University of Murcia, Murcia, Spain; 3 Department of Endocrinology and Metabolism, Academic Medical Center, University of Amsterdam, Amsterdam, the Netherlands; Rutgers University, UNITED STATES

## Abstract

Thyroid dysfunction is associated with changes in coagulation. The aim of our study was to gain more insight into the role of thyroid hormone in coagulation control. C57Black/6J mice received a low-iodine diet and drinking water supplemented with perchlorate to suppress endogenous triiodothyronine (T_3_) and thyroxine (T_4_) production. Under these conditions, the impact of exogenous T_3_ on plasma coagulation, and hepatic and vessel-wall-associated coagulation gene transcription was studied in a short- (4 hours) and long-term (14 days) setting. Comparing euthyroid conditions (normal mice), with hypothyroidism (conditions of a shortage of thyroid hormone) and those with replacement by incremental doses of T_3_, dosages of 0 and 0.5 μg T_3_/mouse/day were selected to study the impact of T_3_ on coagulation gene transcription. Under these conditions, a single injection of T_3_ injection increased strongly hepatic transcript levels of the well-characterized T_3_-responsive genes deiodinase type 1 (*Dio1*) and *Spot14* within 4 hours. This coincided with significantly reduced mRNA levels of *Fgg*, *Serpinc1*, *Proc*, *Proz*, and *Serpin10*, and the reduction of the latter three persisted upon daily treatment with T_3_ for 14 days. Prolonged T_3_ treatment induced a significant down-regulation in factor (*F*) *2*, *F9* and *F10* transcript levels, while *F11* and *F12* levels increased. Activity levels in plasma largely paralleled these mRNA changes. *Thbd* transcript levels in the lung (vessel-wall-associated coagulation) were significantly up-regulated after a single T_3_ injection, and persisted upon prolonged T_3_ exposure. Two-week T_3_ administration also resulted in increased *Vwf* and *Tfpi* mRNA levels, whereas *Tf* levels decreased. These data showed that T_3_ has specific effects on coagulation, with *Fgg*, *Serpinc1*, *Proc*, *Proz*, *Serpin10* and *Thbd* responding rapidly, making these likely direct thyroid hormone receptor targets. *F2*, *F9*, *F10*, *F11*, *F12*, *Vwf*, *Tf* and *Tfpi* are late responding genes and probably indirectly modulated by T_3_.

## Introduction

Abnormalities of blood coagulation are common in patients with thyroid dysfunctions. In general, hyperthyroidism is associated with a hypercoagulable state and increased risk for venous thrombosis [1,2]. In hypothyroidism, the severity of the disorder determines whether the coagulation profile is shifted towards an anticoagulant or procoagulant state as subclinical hypothyroidism is associated with hypercoagulation whereas patients with overt hypothyroidism have a bleeding tendency [3,4].

The mechanism of action of thyroid hormones and thyroid hormone receptors has been well established in a number of metabolic processes, including lipoprotein homeostasis, cell proliferation and mitochondrial respiration [5–7]. However, experimental evidence regarding the mechanisms by which thyroid hormones modulate blood coagulation is more limited, and consists mainly of *in vitro* data showing that triiodothyronine (T_3_) is able to modulate transcript levels of *FGG*, factor (*F*) *2*, *F10* and *SERPINC1*, genes encoding fibrinogen-γ, FII, FX and antithrombin respectively [8–10]. *In vivo* studies with thyroid hormone receptor knock-out mice identified *Fgg* to be modulated by T_3_, whereas T_3_ administration in thyroidectomized rats was able to increase *Fga* (encoding fibrinogen-α), *F2* and *F10* mRNA levels [6,10].

Here, we used an *in vivo* approach to gain a deeper insight into the modulatory role of thyroid hormone on coagulation gene transcription and plasma levels by comparing euthyroid mice (normal chow-fed mice), with mice in which endogenous thyroid hormone production was suppressed, and with mice replaced with the active thyroid hormone, *i*.*e*. T_3_. This approach enabled identification of the specific response to T_3_ at relatively low dosage. Our data demonstrate that T_3_ effects can be both immediate and late by controlling directly and indirectly the transcription of coagulation genes.

## Material and Methods

### Animal experiments

Eight-week-old C57Black/6J male mice were purchased from Charles River (Maastricht, the Netherlands) and received either normal chow diet and drinking water (normal conditions), or received a low iodine diet (ICN Biomedicals, Inc., Aurora, OH, US) while drinking water was supplemented with 1% (wt/vol) potassium perchlorate (Sigma-Aldrich Chemie B.V. Zwijndrecht, The Netherlands) to suppress endogenous thyroid hormone production. 3,3′,5-Triiodo-L-thyronine sodium salt (T_3_; Sigma-Aldrich Chemie B.V. Zwijndrecht, The Netherlands) stocks of 1 mg/mL were prepared in 4 mM sodium hydroxide and stored at 4°C until use. Before injections, the T_3_ stock was diluted (0–10 μg T_3_ /200 μL) in phosphate buffered saline (PBS) supplemented with 0.02% bovine serum albumin with a final concentration of 0.2 mM sodium hydroxide. To determine the effects of a prolonged T_3_ exposure on transcription as well as plasma levels of coagulation factors, mice received a daily intraperitoneal injection of 200 μL T_3_ for 14 days with the different concentrations of T_3_ (0, 0.05, 0.1, 0.5, 1 and 10 μg T_3_ /200 μL). To determine which factors are rapidly modulated by T_3_, a single dose of either 0.5 μg T_3_/mouse or vehicle (PBS supplemented with 0.02% bovine serum albumin with a final concentration of 0.2 mM sodium hydroxide) was administered for 4 hours. After the last administration of either daily doses or single injection, the experimental mice, and normal chow-fed animals (euthyroid controls) were anesthetized by an intraperitoneal injection with a mixture of ketamine (100 mg/kg), xylazine (12.5 mg/kg) and atropine (125 μg/kg) after which the abdomen was opened by a midline incision and a blood sample on sodium citrate (final concentration 0.32%) was drawn from the inferior cava vein. Platelet-poor plasma was obtained and stored at -80°C until use. The liver was isolated and weighed, and part of the left liver lobule and the lungs were snap-frozen for mRNA analyses. All experimental procedures were approved by the animal welfare committee of the Leiden University.

### Plasma analyses

Plasma triiodothyronine (T_3_) and thyroxine (T_4_) levels were measured with in-house radioimmunoassays as previously described [11]. Plasma alanine aminotransferase (ALT), aspartate aminotransferase (AST) and alkaline phosphatase (ALP) levels were determined using routine clinical chemistry assays. Plasma FII and FX activity were analyzed by means of chromogenic substrate conversion, FVII activity was evaluated using the commercially available Biophen FVII kit (Hyphen Biomed, Nodia Bv, Amsterdam, The Netherlands) and activity levels of FVIII, FIX, FXI, FXII were measured in APTT based assays [12]. Plasma fibrinogen and protein C antigen levels were assessed with a commercial murine ELISA kit from Affinity Biologicals and an in-house ELISA using antibodies from Haematologic Technologies Inc, respectively. Antithrombin activity was measured by means of the Coamatic Antithrombin kit (Chromogenix Werfen Benelux, Voorschoten, The Netherlands). For all plasma assays of individual coagulation factors, pooled normal mouse plasma was used to generate standard curves and the vehicle-treated group was subsequently set as a reference (100%).

Global coagulability of the plasma was determined by measuring the activated partial thromboplastin time (APTT) using the STA Neoplastine Plus reagent on the STart 4 analyzer (Diagnostica Stago, Leiden, The Netherlands). The prothrombin time (PT) was determined with the Simple Simon PT system (Zafena, Leiden, The Netherlands) and thrombin generation was assessed by means of the Calibrated Automated Thrombogram, using 5 pM tissue factor (Thrombinoscope, Maastricht, The Netherlands) to trigger 1:6 diluted mouse plasma. Thrombin generation was measured on the Fluoroskan Ascent reader (Thermo Scientific, Bleiswijk, The Netherlands) and the curves and area under the curve (endogenous thrombin potential; ETP) were calculated using the Thrombinoscope software.

### RNA isolation and real-time RT-PCR

Individual liver (20–30 mg) and lung samples (40–50 mg), as a substitute for the vessel wall, were homogenized in RNAzol (Bio-Connect, Huissen, The Netherlands) and RNA isolation and cDNA synthesis was performed as previously described [12]. Quantitative real-time PCR using SybrGreen (Life Technologies, Bleiswijk, The Netherlands) and gene-specific primers (S1 Table) was performed on the ABI Prism 7900 HT Fast Real-Time PCR System from Life Technologies. Data were analyzed using the accompanying Sequence Detection System software and the comparative threshold cycle method with β-actin as an internal control was used for quantification and normalization. Vehicle-treated animals were set as a reference and the ΔC_t_ values of the individual samples were related to the mean ΔC_t_ of the reference group.

### Statistical analyses

Statistical differences were calculated by non-parametric Mann-Whitney U test using GraphPad Prism 6 software (GraphPad Software Inc., San Diego, CA, US). A p-value of <0.05 was considered to be statistically significant.

## Results

### Dose-finding study: 0.5 μg/day is the optimal T_3_ dose to induce changes in coagulation

A dose-finding study was performed in which the effects of thyroid hormone deprivation and subsequent treatment with incremental doses of T_3_ (from 0–10 μg/mouse/day; n = 6 per group) on a representative panel of coagulation factors was studied, allowing selection of an optimal T_3_ dose for further evaluation of the effects T_3_ on coagulation.

Two weeks feeding a low iodine diet and drinking water supplemented with potassium perchlorate successfully reduced endogenous plasma thyroid hormone levels as compared to normal mice (-75% and -80% for T_3_ and T_4_, respectively; Table 1). This coincided with statistically significant increases in body weight, liver weight and plasma aspartate transaminase, while plasma alkaline phosphatase levels significantly decreased as compared to euthyroid mice (Table 1). As expected deprivation of thyroid hormone coincided with a dramatic significant reduction of hepatic transcript levels of *Dio1*, a well-characterized thyroid hormone response gene (Table 2). For a selected panel of coagulation genes, thyroid hormone deprivation resulted in marked increases in coagulation transcript levels as compared to normal conditions. For the liver, significant increases were observed for *Fgg*, *F2*, *Serpinc1*, *Proc* (encoding protein C), and *Pros1* (encoding protein S) (See Table 2). For the lung, where highly vascularized tissue serves here as a substitute for the vessel wall, significant increases in transcript levels were observed for all coagulation genes selected *i*.*e Vwf* (encoding von willebrand factor), *Thbd* (encoding trombomodulin), and *Procr* (encoding endothelial protein C receptor) (See Table 2). For *Fgg*, *F2* and *F12* changes in hepatic transcript levels were paralleled by significant changes in fibrinogen-γ, FII, and FXII plasma protein activity levels (See Table 3).

**Table 1:** General and plasma parameters upon increasing doses of T_3_.

T_3_ (μg/mouse/day)	EM	0	0.05	0.1	0.5	1	5	10
**Body Weight (g)**	24.0±0.4	26.2±0.3*	26.4±0.5*	26.5±1.0*	28.6±0.7*^‡^	27.9±0.4*^‡^	27.7±0.3*^‡^	27.7±0.5*^‡^
**Liver Weight (g)**	0.79±0.02	1.51±0.07*	1.30±0.06*^‡^	1.28±0.09*	1.24±0.04*^‡^	1.21±0.03*^‡^	1.09±0.01*^‡^	1.10±0.02*^‡^
**T**_**3**_ **(nmol/L)**	1.22±0.04	0.31±0.01*	1.46±0.10^‡^	2.09±0.12*^‡^	4.99±0.50*^‡^	8.78±1.84*^‡^	26.21±3.18*^‡^	51.20±5.84*^‡^
**T**_**4**_ **(nmol/L)**	60.7±4.2	12.0±1.5*	9.3±0.8*	8.8±0.6*	9.6±1.7*	11.8±0.7*	14.0±1.0*	10.0±0.7*
**ALT (U/L)**	20.1±2.2^**#**^	37.5±9.6	30.8±6.3	24.2±3.3	20.1±0.1	30.0±7.1	55.0±7.6*	70.0±14.9*
**AST (U/L)**	88.3±4.3^**#**^	98.7±21.9*	113.3±7.2*	71.7±8.0*	71.0±3.4*	93.3±13.1	134.2±18.4*	156.0±24.0*
**ALP (U/L)**	26.1.±2.0^**#**^	61.3±1.0*	72.5±3.4*^‡^	81.7±6.0^‡^	140.0±15.1^‡^	166.7±12.3	282.5±23.1*^‡^	313.0±11.3*^‡^

ALT: alanine aminotransferase; AST: aspartate aminotransferase; ALP: alkaline phosphatase; EM: Euthyroid (normal) mice. Hashtag: For the normal euthyroid mice only, plasma liver enzymes (ALT, AST and ALP) were obtained from different group of identical normal euthyroid mice. Data are presented as mean±SEM. Asterisks: For p-values comparing data of EM (set as a reference) *vs* each column of increasing doses of T_3_ (*p<0.05). Daggers: For p-values comparing data of (0) vehicle-treated group (set as a reference) *vs* each column of increasing doses of T_3_ **(** ^‡^p<0.05).

**Table 2:** Hepatic and vessel-wall-associated target transcripts levels.

T_3_ (μg/mouse/day)		EM	0	0.05	0.1	0.5	1
**Hepatic Transcript Levels**	***Dio1***	73.4 (66.9–80.4)	1 (0.83–1.20)*	24.2 (19.3–30.3)*^‡^	76.1 (58.4–88.9)^‡^	181 (148–222)*^‡^	191 (157–232)*^‡^
	***Fgg***	0.85 (0.79–0.90)	1 (0.96–1.04)	1.03 (0.88–1.20)	1.07 (0.97–1.19)	0.56 (0.49–0.63)*^‡^	0.47 (0.42–0.53)*^‡^
	***F2***	0.76 (0.72–0.80)	1 (0.93–1.07)*	0.70 (0.60–0.82)	0.88 (0.84–0.92)	0.62 (0.56–0.68)^‡^	0.64 (0.57–0.71)^‡^
	***F12***	1.23 (1.19–1.28)	1 (0.90–1.11)	1.14 (1.04–1.25)	1.20 (1.06–1.35)	1.23 (1.14–1.32)	1.01 (0.89–1.15)
	***Serpinc1***	0.80 (0.75–0.86)	1 (0.97–1.04)*	0.86 (0.79–0.94)	0.97 (0.95–1.00)*	0.79 (0.76–0.82)^‡^	0.84 (0.75–0.95)
	***Proc***	0.51 (0.46–0.56)	1 (0.95–1.05)*	0.82 (0.76–0.88)*	0.82 (0.78–0.86)*^‡^	0.57 (0.53–0.62)^‡^	0.56 (0.51–0.61)^‡^
	***Pros1***	0.77 (0.71–0.84)	1 (0.94–1.06)*	0.91 (0.85–0.98)	1.05 (0.99–1.11)*	0.84 (0.81–0.87)	0.82 (0.74–0.91)
**Vessel-Wall-Associated Transcript Levels**	***Vwf***	0.70 (0.63–0.77)	1 (0.95–1.05)	1.01 (0.97–1.05)*	1.00 (0.94–1.05)*	0.97 (0.92–1.03)	0.98 (0.93–1.03)*
	***Tf***	0.32 (0.27–0.39)	1 (0.94–1.07)*	0.79 (0.75–0.83)*^‡^	0.72 (0.67–0.77)*^‡^	0.66 (0.59–0.73)*^‡^	0.68 (0.61–0.76)*^‡^
	***Thbd***	0.60 (0.49–0.73)	1 (0.93–1.07)*	1.64 (1.54–1.73)*^‡^	1.54 (1.43–1.66)*^‡^	1.61 (1.45–1.78)*^‡^	1.54 (1.43–1.66)*^‡^
	***Procr***	0.59 (0.53–0.64)	1 (0.94–1.07)*	0.86 (0.76–0.97)*	0.75 (0.69–0.83)	0.81 (0.71–0.91)*	0.87 (0.80–0.96)*

EM: Euthyroid (normal) Mice. Data are presented as mean and confidence intervals values. Asterisk: For p-values comparing data of EM (set as a reference) *vs* each column of increasing doses of T_3_ (*p<0.05). Daggers: For p-values comparing data of (0) vehicle-treated group (set as a reference) *vs* each column of increasing doses of T_3_ **(** ^‡^p<0.05).

**Table 3:** Plasma levels of coagulation factors upon increasing doses of T_3_.

T_3_ (μg/mouse/day)	EM	0	0.05	0.1	0.5	1	5	10
**FVIII**	94.7±1.9	87.8±6.9	100.1±6.5	109.8±9.1	92.0±5.5	78.6±5.2	75.7±7.0	76.1±6.6
**FIX**	92.9±1.3	96.5±2.8*	88.4±3.6*	95.7±3.1*	89.0±3.3*	92.2±3.5*	84.6±3.6*^‡^	88.6±4.1*
**FXII**	105.7±2.5	81.0±2.5*	95.1±2.6^‡^	99.3±2.9*^‡^	102.8±2.5^‡^	101.5±5.0^‡^	96.2±3.6*^‡^	104.1±2.3*^‡^
**FII**	90.6±5.3	126.1±2.1	92.4±3.5*^‡^	93.4±2.5*^‡^	74.9±4.4*^‡^	63.7±3.9*^‡^	60.6±3.5*^‡^	63.5±2.9*^‡^
**FX**	98.4±1.4	104.0±2.2	86.8±1.9*^‡^	91.6±2.8^‡^	82.2±3.3*^‡^	74.1±7.5*^‡^	68.5±2.1*^‡^	71.1±3.5*^‡^
**Antithrombin**	107.6±1.2	113.7±2.5	88.0±3.5*^‡^	94.4±3.8*^‡^	74.6±3.8*^‡^	83.4±2.2*^‡^	79.8±1.9*^‡^	75.7±3.3*^‡^
**Fibrinogen**	94.2±6.7	101.2±5.3*	98.8±3.2*	92.8±16.0	60.0±7.4*^‡^	50.8±4.6*^‡^	49.3±2.3*^‡^	40.5±0.8*^‡^

EM: Euthyroid (normal) Mice. Data are presented as mean±SEM. Asterisks indicate p-values comparing data of EM (set as a reference) *vs* each column of increasing doses of T_3_ (*p<0.05). Daggers indicate p-values comparing data of (0) vehicle-treated group (set as a reference) *vs* each column of increasing doses of T_3_ **(** ^‡^p<0.05).

Subsequent treatment of hypothyroid mice with incremental doses of T_3_ resulted in dose-dependent increase in plasma T_3_ levels from 0.31±0.01 nmol/L for vehicle-treated mice to a maximum of 51.2±5.8 nmol/L for mice treated with 10 μg T_3_/day (p<0.001). T_4_ levels were low (range 8.8±0.6–14.0±1.0 nmol/L) and did not differ between treatment groups (Table 1).

Increasing T_3_ resulted in a dose-dependent increase in body weight up to a dose of 0.5 μg/day (weight gain during 14 days: 0.18±0.26 g *vs*. 2.26±0.36 g, p<0.01; Table 1), whereas the liver weight decreased dose-dependently (see Table 1). The circulating liver enzymes ALT and AST levels were not affected in T_3_-treated mice as compared to vehicle treatment, whereas ALP levels differed significantly (see Table 1). However, administration of increasing amounts of T_3_ (from 0.5 μg/day upwards) resulted in relatively modest increased in ALT and AST levels (see Table 1). As doses of 5 and 10 μg T_3_/day had higher liver enzyme levels, these doses were excluded from mRNA analyses. In the range from 0–1 μg T_3_/mouse/day, hepatic *Dio1* transcript levels were dose-dependently up-regulated, opposite to what was observed upon thyroid hormone deprivation, as expected (Table 2). Hepatic *Fgg* and *F2* transcripts were reduced, and were significantly different in mice treated with 0.5 μg T_3_/day as compared to vehicle-treated animals, whereas the transcript levels of *F12* and *Serpinc1* were not significantly affected (Table 2). For transcript levels of the vessel-wall-associated coagulation factors, increasing T_3_ doses resulted in a dose-dependent decrease of *Tf* mRNA levels and a rise in *Thbd* levels, while *Vwf* and *Epcr* levels were not affected (Table 2).

Plasma levels of fibrinogen antigen, FII, FX and antithrombin activity levels were dose-dependent decreasing, whereas FXII activity was increased, and as compared to the vehicle treatment these effects were significantly different from a dose of 0.5 μg upwards (Table 3).

Based on these data, for further studies, comparisons were made between mice deprived of endogenous thyroid hormone and will receive either vehicle, short-term (4 hour) or long-term (14 day) exposure to 0.5 μg T_3_/mouse/day. The 0.5 dose should be considered a pharmacological T_3_ dose able to induce a condition of hyperthyroidism, and is approximately 10-fold the dose required to establish euthyroid / physiological levels of T_3_ (i.e. established by a dose of 0.05 μg T_3_/mouse/day (Table 1))". Using the thyroid deprived background solely allows identification of effects that are specific to T_3_ (and corrects for possible effect or other components of the low-iodine diet, repetitive injection and aberrant drinking water (perchlorate supplemented)).

### Single T_3_ dose: limited effects upon transcription of coagulation genes

Twelve mice with a suppressed thyroid hormone production per group were treated with either the vehicle (0 μg T_3_/mouse) or a single T_3_ injection (0.5 μg/mouse). We examined the hepatic transcript levels 4 hours later in order to determine whether T_3_ is able to directly affect transcription of coagulation genes, *i*.*e*. via a direct interaction between the ligand-bound thyroid hormone receptor and the promoter region of coagulation genes.

**Fig 1:**
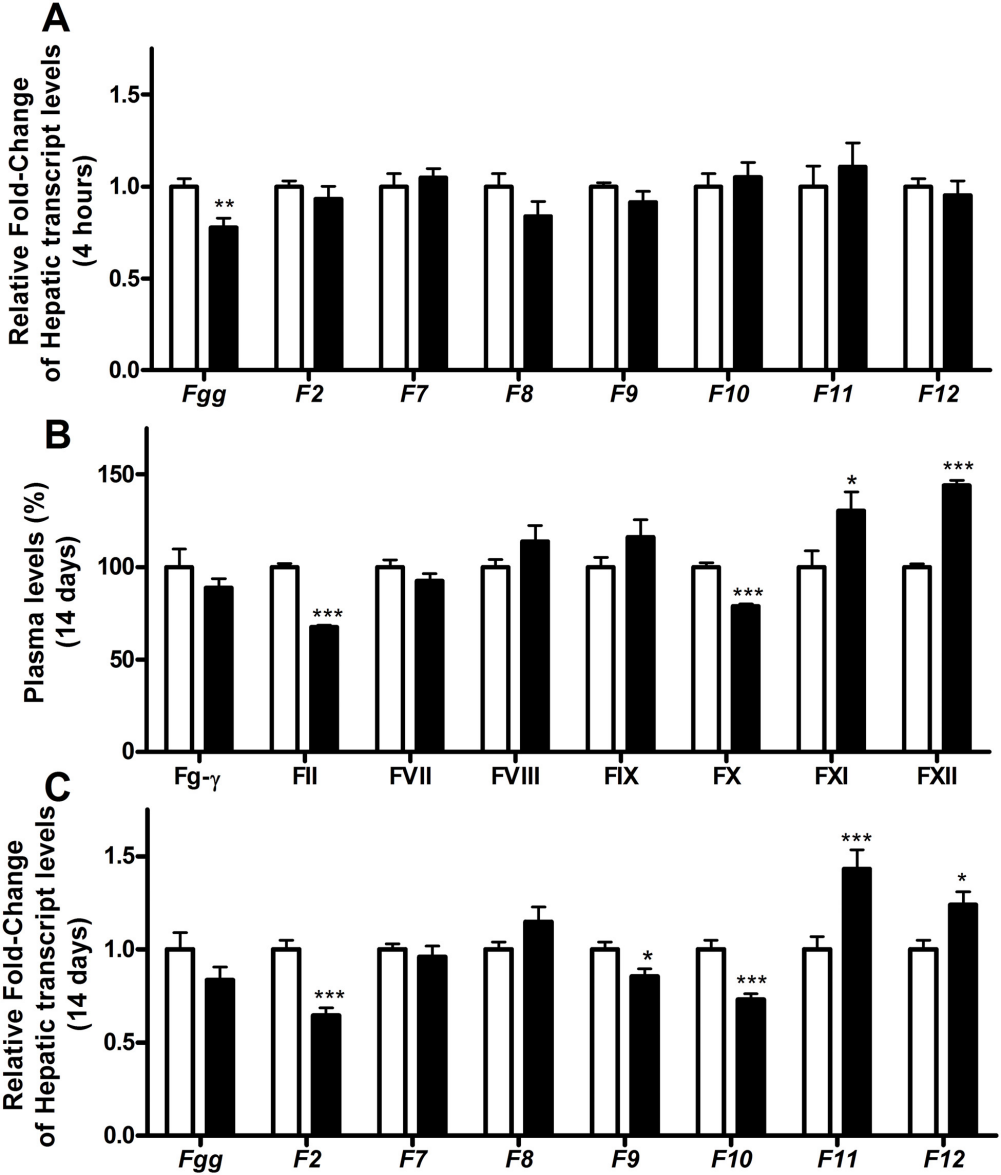
Hepatic transcript levels and plasma levels of procoagulant coagulation factors. Hepatic transcript levels (A and C) and plasma levels (B) of procoagulant coagulation factors in mice treated with 0 μg T_3_ (white bars) or 0.5 μg T_3_ (black bars). Panel A shows the T_3_-induced changes in hepatic transcript levels 4 hours after a single T_3_ injection. Panels B and C show plasma levels and T_3_-induced changes in hepatic transcripts for 14 days, respectively. Data are presented as mean with the error bar representing the calculated maximum expression level (panels A and C) or mean±SEM (standard error of the mean) (panel B) of n = 12 mice per group, with the vehicle-treated group set as a reference. Relative expression levels (A and C) were compared using the comparative threshold cycle method with ß-actin as internal control. *p<0.05, **p<0.01, and ***p<0.001 as compared to vehicle-treated mice. Fg-γ: Fibrinogen-γ plasma levels.

**Fig 2:**
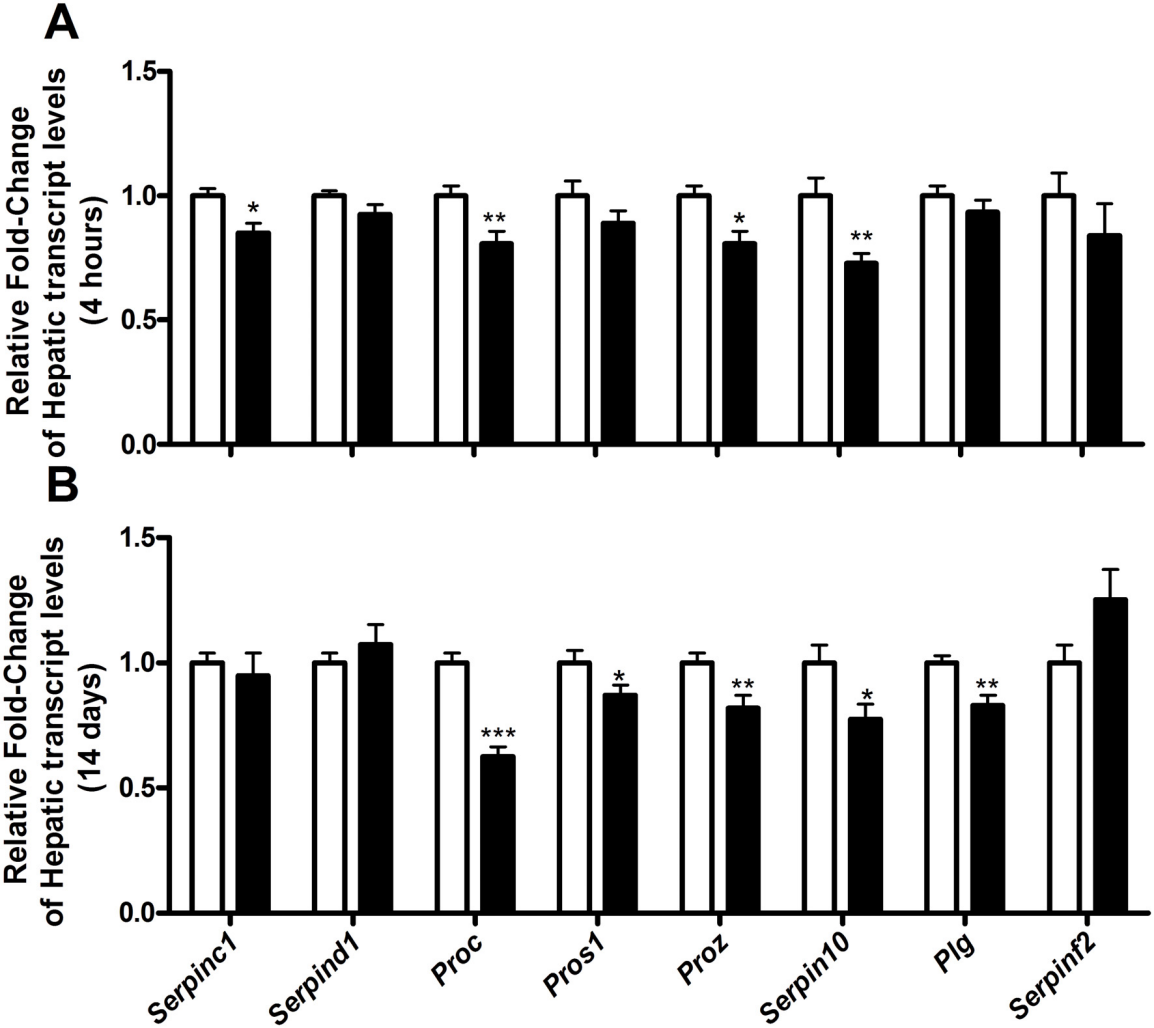
Hepatic transcript levels of anticoagulant and fibrinolytic factors. Hepatic transcript levels of anticoagulant and fibrinolytic factors in mice treated with 0 μg T_3_ (white bars) or 0.5 μg T_3_ (black bars) given a single dose (A) or for 14 days (B). Data are presented as mean with the error bar representing the calculated maximum expression level of n = 12 mice per group and the vehicle-treated group set as a reference. Relative expression levels were compared using the comparative threshold cycle method with ß-actin as internal control. *p<0.05, **p<0.01, and ***p<0.001 as compared to vehicle-treated mice.

**Fig 3:**
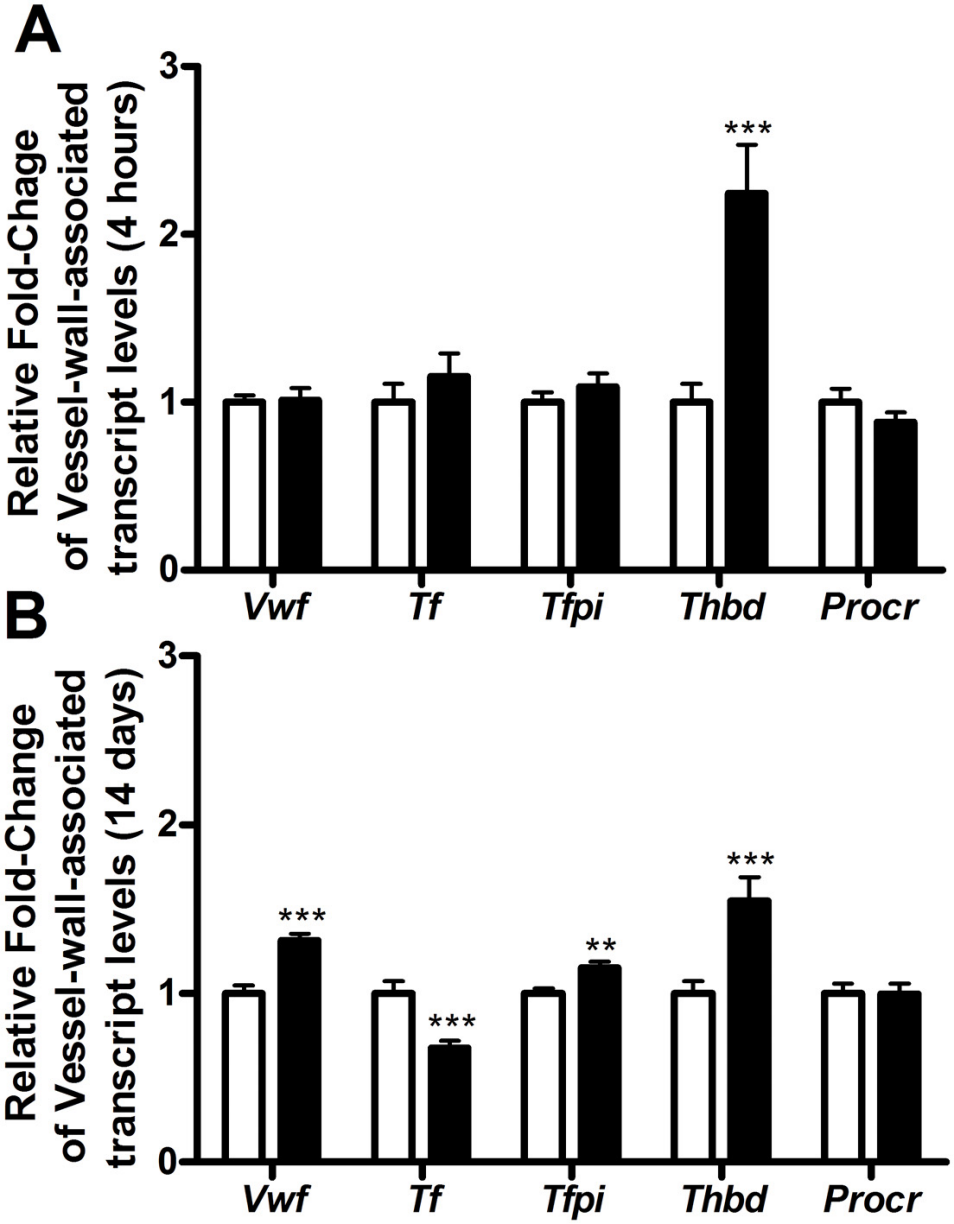
Transcript levels of vessel-wall-associated coagulation factors. Transcript levels of vessel-wall-associated coagulation factors measured in mice treated with 0 μg T_3_ (white bars) or 0.5 μg T_3_ (black bars) given a single dose (A) or for 14 days (B). Data are presented as mean with the error bar representing the calculated maximum expression level of n = 12 mice per group and the vehicle-treated group set as a reference. Relative expression levels were compared using the comparative threshold cycle method with ß-actin as internal control. *p<0.05, **p<0.01, and ***p<0.001 as compared to vehicle-treated mice.

Already after these 4 hours, the hepatic transcript levels of the canonical T_3_-responsive genes *Dio1* and *Spot14* were increased (1±0.18 *vs*. 136.4±29.3 and 1±0.36 *vs*. 5.80±1.26, respectively). Under these conditions, hepatic transcript levels of *Fgg*, *Serpinc1*, *Proc*, *Proz* and *Serpin10* were significantly reduced (Figs 1A and 2A). A single T_3_ dose was not able to induce significant changes in *F2*, *F10*, *F11*, *F12*, *Pros1*, and *Plg* (encoding plasminogen) transcript levels (Figs 1A and 2A). Transcript levels of most vessel-wall-associated clotting factors in the lung remained unaffected but *Thbd* transcript levels increased markedly after a single T_3_ injection (Fig 3A). Remarkably, this unique dosage has no effect on the body and liver weight. Since the transcriptional machinery evolved T_3_-responsive genes takes time, changes in plasma markers of coagulation and fibrinolysis cannot be detected after 4 hours of treatment.

### Prolonged T_3_ treatment: widespread effects upon transcription of coagulation genes

In order to determine T_3_ effects in the transcription of coagulation factors in a long-term, twelve mice with a suppressed thyroid hormone production per group were treated with either the vehicle (0 μg T_3_/mouse/day) or a dose of 0.5 μg T_3_/mouse/day for 2 weeks. This treatment regime again as in the dose finding study resulted in an increase in body weight (weight gain: 0.24±0.15 g *vs*. 2.12±0.20 g, p<0.001) and a reduction in liver weight (0.84±0.02 g *vs*. 0.74±0.02 g, p<0.001). As expected, T_3_ levels were significantly higher in T_3_-treated mice (5.64±0.21 nmol/L *vs*. 0.33±0.01 nmol/L, p<0.001), whereas T_4_ levels were low and did not differ between treatment groups (7.2±0.2 nmol/L *vs*. 8.7±1.0 nmol/L).

Fig 1B shows that, in line with the dose-finding study, plasma FII and FX activity levels decreased upon T_3_ treatment, while FXII levels increased. FVIII and FIX levels were not significantly affected. In addition, FVII levels did also not differ between vehicle- and T_3_-treated animals, whereas FXI activity levels increased due to T_3_ administration. Plasma antithrombin and protein C antigen levels were both significantly lower in T_3_-treated mice (100±1.6% vs. 92.6±1.2%, p<0.001 for antithrombin; 100±4.0% vs. 85.9±3.1%, p = 0.009 for protein C).

**Fig 4:**
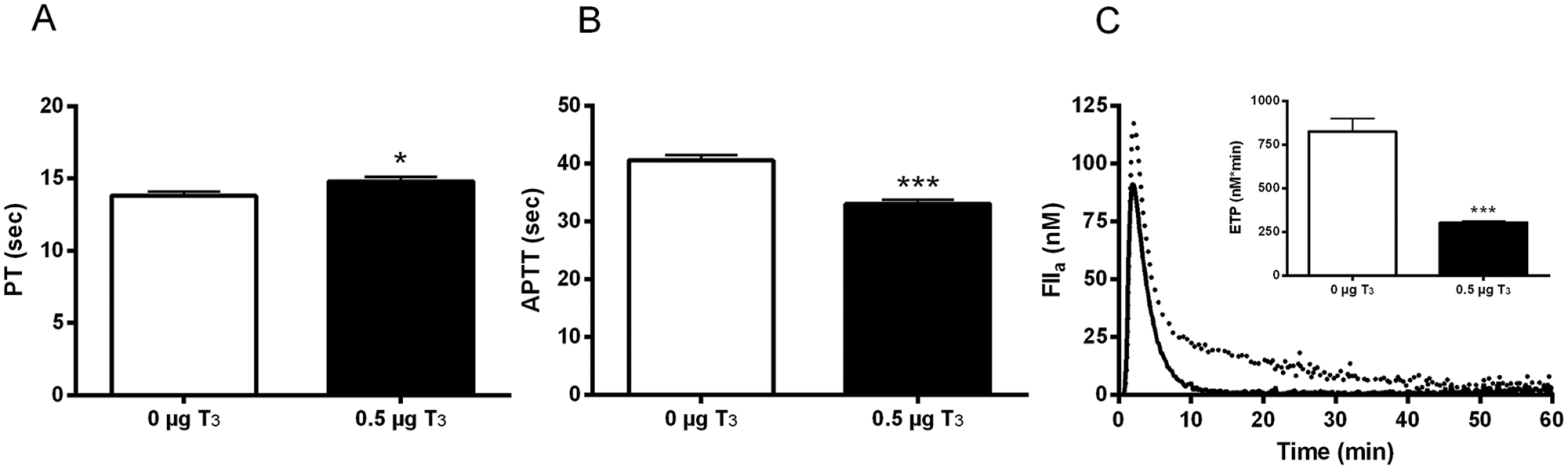
Global coagulability of the plasma assays. Plasma prothrombin time (PT; A), activated partial thromboplastin time (APTT; B) and averaged thrombin generation curves with the resulting endogenous thrombin potential values (ETP; C) for mice treated with 0 μg T_3_ (white bars, dotted line) or 0.5 μg T_3_ (black bars, solid line) for 14 days. Data are presented as mean±SEM of n = 12 mice per group. *p<0.05 and ***p<0.001 as compared to vehicle-treated mice.

Prolonged T_3_ treatment was able to induce significant changes in plasma levels of FII, FX, FXI, and FXII (see Fig 1B). Consistent with the lower FII and FX levels upon T_3_ treatment, the PT was longer in T_3_-treated animals and the increased FXI and FXII levels resulted in a shorter APTT (Fig 4A and 4B). To assess the overall plasma coagulability, thrombin generation was measured, showing a lower endogenous thrombin potential in mice treated with 0.5 μg T_3_/day as compared to vehicle-treated mice, which was mainly due to a lower peak height and an earlier onset of the inhibition of thrombin activity (Fig 4C).

With the exception of FIX levels, T_3_-induced effects on plasma proteins were completely paralleled by changes in hepatic transcript levels (See Fig 1B and 1C). The transcript levels of anticoagulant genes and the fibrinolytic factors were determined and presented in Fig 2B. Strikingly, T_3_ administration caused only significant decreases (no increases) in mRNA levels of anticoagulant factors which included *Proc*, *Pros1*, *Proz*, *Serpin10*, and *Plg* (plasminogen). These decreases were comparable to the reduction observed after a single dose of T_3_, although the immediate decrease of *Pros1* mRNA after a single dose of T_3_ did not reach statistical significance (Fig 2A and 2B). While *Fgg* and *Serpinc1* were significantly reduced 4 hours after a single T_3_ injection and in the dose-finding study, these effects were not apparent after prolonged T_3_ exposure. (Figs 1A *vs* 1C and 2A *vs* 2B, respectively)

In line with the dose-finding study, *Tf* transcript levels in the lung were reduced and *Thbd* levels were up-regulated upon two-week T_3_ exposure (Fig 3B). In addition, *Vwf* levels showed a significant increase, as well as the mRNA levels of *Tfpi*, whereas *Procr* levels remained unaffected by T_3_ treatment.

These data show that prolonged T_3_ exposure alters the plasma coagulation profile by controlling hepatic transcript levels of coagulation genes. In addition, transcription of vessel-wall-associated coagulation genes, measured at the level of the lung, can also be modulated by T_3_ administration.

## Discussion

Evidence is accumulating that overt hypothyroidism and hyperthyroidism are associated with changes in the haemostatic balance, which translates to either a bleeding tendency or an increased thrombosis risk [1–4]. However, the underlying mechanism how thyroid hormone can modulate coagulation is largely unknown. In the present study, we demonstrate that intraperitoneal administration of triiodothyronine (T_3_) to hypothyroid mice modulates transcription of both hepatic and vessel-wall-associated coagulation factors. *Fgg*, *Serpinc1*, *Proc*, *Proz*, *Serpin10* and *Thbd* responded rapidly upon a single T_3_ injection. On the other hand, factors *2*, *9*, *10*, *11*, *12*, *Vwf*, *Tf*, and *Tfpi* were only modulated after a prolonged T_3_ exposure, *i*.*e*. 14 days. Although analyzed for a limited set of liver-derived coagulation factors, the changes in transcript levels were largely paralleled by changes in plasma levels. Based on these observations, we conclude that T_3_ has immediate and late effects on coagulation in mice.

Our data are in line with observations by Flores-Morales et al., who showed that T_3_ can have both immediate and late effects on mouse hepatic gene transcription, and that these effects can be either up- or down-regulated [6]. The coagulation factors that responded within 4 hours after injection are likely to be directly regulated by thyroid hormone, via interaction with the thyroid hormone receptor and corresponding response elements in the promoter region of coagulation genes. Surprisingly, a number of these genes are directly negatively regulated with the exception of thrombomodulin. Although it is known that transcriptional suppression is a common action of thyroid hormones [5–7], the mechanisms underlying this negative regulation are poorly understood and may involve binding of co-repressors like NCOR1 or post-transcriptional microRNA binding. Some studies provide new insight into the role of miRNAs in mediating thyroid hormone regulation of gene expression [13], while chromatin remodelling and DNA methylation may also play a role in transcriptional suppression [14,15].

At present we do not provide direct evidence whether the immediate action of T_3_ on coagulation gene transcription also involves thyroid hormone receptors. To demonstrate this, it would require to follow our experimental design and methodology presented here, using mice lacking *TRα1* ^−/−^ [16], *TRβ* ^−/−^ [17] or both *TRα1* ^−/−^ *β* ^−/−^ [18], or specific thyroid receptor antagonists in normal mice [19–21]. Such experiments would shine a light on the direct role of thyroid hormone receptors in coagulation gene transcription control and which receptor subtype is involved.

For the genes that require a prolonged T_3_ exposure to evoke a clear transcriptional response, an indirect modulation is more likely which can involve intermediate transcription factors additional to the thyroid hormone receptor. Despite the fact that there are many intermediates possible, we hypothesized that the hepatic nuclear factor 4α (HNF4α) would be a good candidate since it is known that thyroid hormone can increase HNF4α expression [22], and the well-established HNF4α targets coagulation FXI and FXII [23] are clearly up-regulated upon prolonged T_3_ exposure. However, the role of HNF4α was not supported by the data as we observed a 20% decrease in hepatic mRNA levels in livers of T_3_ mice (data not shown).

Our observations in lung samples as a substitute for the vasculature clearly indicate that also the (micro)vasculature with its vessel-wall-associated coagulation factors is responsive to T_3_, as the levels of most factors were affected after prolonged T_3_ administration. Interestingly, *Thbd* appeared to be an immediate responder and could be induced by a T_3_ dose as low as 0.05 μg/mouse/day, indicating that *Thbd* transcription is highly sensitive to T_3_. Although we were not able to determine *Thbd* protein levels, it has been reported that patients with hyperthyroidism have increased levels of circulating soluble thrombomodulin [23,24].

In humans, hyperthyroidism is associated with an increased risk for thrombosis, while we showed that thyroid hormone administration in mice results in an increase in prothrombin time and a decrease in the endogenous thrombin potential, which point towards a bleeding tendency instead of a thrombotic tendency. These results can at least be partially explained by the observed decreases in plasma FII and FX levels. On the other hand, the APTT was shorter due to the increased FXI and FXII levels, suggesting a thrombosis-prone condition, which is more in line with what would be expected based on human observations. Although this *in vivo* study shows the value of mice in mechanistic studies, these findings also indicate that the use of mice in studying thyroid disorders in human-like coagulopathies, *i*.*e*. bleeding or thrombosis, faces limitations.

In conclusion, our study demonstrates that T_3_ administration to hypothyroid mice has widespread effects on transcription of hepatic and vessel-wall-associated coagulation genes [measured at the level of the lung]. Furthermore, we identified both immediate and late responding coagulation genes, suggesting that T_3_ can either directly or indirectly control transcription. In addition, the transcriptional changes resulted in altered plasma levels of a panel of coagulation factors. We believe that this mouse study contributes to a better understanding of the relation between thyroid dysfunctions and coagulation disorders in human.

## Supporting Information

S1 Table QPCR primer sequences. Sequence of primers used for qPCR.
(DOCX)
