# Supplementary material for: The Immediate and Late Effects of Thyroid Hormone (Triiodothyronine) on Murine Coagulation Gene Transcription
Source: PLoS One. 2015 May 26;10(5):e0127469. doi: 10.1371/journal.pone.0127469 (PMC4444115; doi:10.1371/journal.pone.0127469)
Supplement: S1 Table — Sequence of primers used for qPCR. (DOCX) [file pone.0127469.s001.docx]

**S1 Table. QPCR primer sequences.** Sequence of primers used for qPCR.

| **Gene** | **Forward primer (5’- 3’)** | **Reverse primer (5’- 3’)** |
| --- | --- | --- |
| ***Actb*** | AGGTCATCACTATTGGCAACGA | CCAAGAAGGAAGGCTGGAAAA |
| ***Dio1*** | TTGCCTCCACAGCCGATT | TCTTAAAAGCCCAGCCATCTG |
| ***Spot14*** | AAGGTGGCTGGCAACGAA | TCGGCCTCCGTTTC |
| ***Fgg*** | TGCTGCCTGCTTTTACTGTTCTC | TCTAGGATGCAACAGTTATCTCTGGTA |
| ***F2*** | GGACGCTGAGAAGGGTATCG | CCCCACACAGCAGCTCTTG |
| ***F7*** | CGTCTGCTTCTGCCTAGA | ATTTGCACAGATCAGCTGCTCAT |
| ***F8*** | CTTCACCTCCAGGGAAGGACTA | TCCACTTGCAACCATTGTTTTG |
| ***F9*** | GCAAAACCGGGTCAAATCC | ACCTCCACAGAATGCCTCAATT |
| ***F10*** | GTGGCCGGGAATGCAA | AACCCTTCATTGTCTTCGTTAATGA |
| ***F11*** | GAAGGATACGTGCAAGGGAGATT | CAAGTGCCAGACCCCATTGT |
| ***F12*** | GGGCTTCTCCTCCATCACCTA | GCAACTGTTGGTTTTGCTTTCC |
| ***Serpinc1*** | TGGGCCTCATTGATCTCTTCA | CCTGCCTCCAGCAACGAT |
| ***Serpind1*** | GAATGGCAATATGTCAGGCATCT | CACTGTGATGGTACTTTGGTGCTT |
| ***Proc*** | GCGTGGAGGGCACCAA | CCCTGCGTCGCAGATCAT |
| ***Pros1*** | GGTGGCATCCCAGATATTTCC | CACTTCCATGCAGCCACTGT |
| ***Proz*** | GCAGCCAGAGTCAGCCTAGCT | CACGCCGGCACAGAAGTC |
| ***PZI*** | TGGCCCTGGAGGACTACTTG | CCATTTTCCTGGTTTTCATATTCTG |
| ***Plg*** | TGACATTGCCCTGCTGAAAC | CAGACAAGCTGGAATGACTTTATCC |
| ***Serpinf2*** | TTCTCCTCAACGCCATCCA | GGTGAGGCTCGGGTCAAAC |
| ***Vwf*** | AGCTGTCAGCCAGGTTTTTCTT | GCAGAGGGCAGGCACCTT |
| ***Tf*** | GGAGGAGCCGCCATTTACA | CTGGCTGTCCAAGGTTTGTGT |
| ***Tfpi*** | CCCAGGCGTCGGGATTA | AATCCACTGTCTGCTGGTTGAA |
| ***Thbd*** | GCGAAATGTTCTGCAATGAAAC | GGCATTCACAAACAGTAGGAGAGTT |
| ***Procr*** | AACCACATCACCACGCAAAA | CCCAGGACCAGTGATGTGTAAG |

*Actb*: gene enconding β-Actin
